# Study of flux pinning mechanism under hydrostatic pressure in optimally doped (Ba,K)Fe_2_As_2_ single crystals

**DOI:** 10.1038/srep23044

**Published:** 2016-03-17

**Authors:** Babar Shabbir, Xiaolin Wang, Y. Ma, S. X. Dou, S. S. Yan, L. M. Mei

**Affiliations:** 1 Spintronic and Electronic Materials Group, Institute for Superconducting and Electronic Materials, Faculty of Engineering, Australian Institute for Innovative Materials, University of Wollongong, North Wollongong, NSW 2522, Australia; 2 Key Laboratory of Applied Superconductivity, Institute of Electrical Engineering, Chinese Academy of Sciences, 2703, Beijing 100190, P. R. China; 3 School of Physics, Shandong University, Shandong, Jinan, 250100, P. R. China

## Abstract

Strong pinning depends on the pinning force strength and number density of effective defects. Using the hydrostatic pressure method, we demonstrate here that hydrostatic pressure of 1.2 GPa can significantly enhance flux pinning or the critical current density (*J*_*c*_) of optimally doped Ba_0.6_K_0.4_Fe_2_As_2_ crystals by a factor of up to 5 in both low and high fields, which is generally rare with other *J*_*c*_ enhancement techniques. At 4.1 K, high pressure can significantly enhance *J*_*c*_ from 5 × 10^5 ^A/cm^2^ to nearly 10^6 ^A/cm^2^ at 2 T, and from 2 × 10^5 ^A/cm^2^ to nearly 5.5 × 10^5 ^A/cm^2^ at 12 T. Our systematic analysis of the flux pinning mechanism indicates that both the pinning centre number density and the pinning force are greatly increased by the pressure and enhance the pinning. This study also shows that superconducting performance in terms of flux pinning or *J*_*c*_ for optimally doped superconducting materials can be further improved by using pressure.

Flux pinning has been a topic of much interest in the field of superconductivity because of its importance for applications and aspects of fundamental physics. This interest stems from the significance of flux pinning for high critical current density (*J*_*c*_) in superconductors, which is the defining property of a superconductor. Generally, various types of random imperfections, such as cold-work-induced dislocations, secondary-phase precipitates, defects induced by high energy ion irradiation, etc., can be used to enhance flux pinning. Unfortunately, it is difficult to discern the maximum potential of a superconductor from these techniques, and the outcomes hold up only to a certain level. Furthermore, the critical current is only enhanced, in most cases, either in low or high fields, but not in both, while degradation of the superconducting critical temperature (*T*_*c*_) is another drawback. For instance, proton irradiation can only enhance flux pinning in high fields by inducing point defects in K:Ba122 1. Similarly, light ion C^4+^ irradiation of Ba122:Ni crystals can only enhance *J*_*c*_ in low fields at high temperatures^2^. High energy particle irradiation can also decrease the critical superconducting temperature (*T*_*c*_) by more than 5 K for cobalt and nickel doped Ba-122 3,4.

As is well known, *J*_*c*_ is mostly limited by weak links (in the case of polycrystalline bulks), and thermally activated flux creep (an intrinsic property) emerges from weak pinning^5^,^6^,^7^,^8^,^9^,^10^,^11^. Strong pinning can be achieved by inducing effective pinning centres with strong pinning force. Our previous results show that *J*_*c*_ is enhanced significantly under hydrostatic pressure in high fields (i.e., over one order of magnitude) in comparison to low fields, along with enhancement of the closely related *T*_*c*_ by more than 5 K in Sr_4_V_2_O_6_Fe_2_As_2_ polycrystalline bulks and NaFe_0.97_Co_0.03_As single crystals^12^,^13^. Until now, however, it has been unclear whether the observed *J*_*c*_ enhancement under pressure is correlated with improved *T*_*c*_ or flux pinning. The primary motivation for the present work is to use optimally doped single crystal samples (which have an unchanged *T*_*c*_ under hydrostatic pressure) to elucidate the contributions of flux pinning to *J*_*c*_ enhancement in Fe-based superconductors. The secondary motivation is to investigate further the contributions from both the pinning centre number density (*N*_*p*_) and the pinning force (*F*_*p*_) to strong pinning.

The argument is as follows: Hydrostatic pressure can induce pinning centres, which, in turn, enhance the pinning force. The total pinning force and the pinning centres are correlated by *F*_*p*_ = *N*_*p*_*f*_*p*_ where *N*_*p*_ is the number density of pinning centres and *f*_*p*_ is the elementary pinning force, defined as the maximum pinning strength of an individual pinning centre, with a value that depends on the interaction of the flux line with the defect. According to the flux pinning theory, strongly interacting defects can contribute to *F*_*p*_ individually, provided that *F*_*p*_ ∝ N_*p*_, and weakly interacting defects can contribute only collectively; the collective theory therefore gives *F*_*p*_ ∝ (*N*_*p*_)^2^ for small defect numbers^14^.

K:Ba122 compound is believed to be the most technologically suitable because of its isotropic nature and high *T*_*c*_, upper critical field (*H*_*c2*_), and *J*_*c*_ values (*J*_*c*_ > 106 A/cm2 at 2 K and 0 T)^15^,^16^,^17^,^18^,^19^. According to the Ginzburg-Landau theory, the depairing current density (*J*_*d*_) is the maximum current density that superconducting electrons can support before de-pairing of Cooper pairs, and is given as


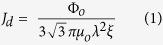


where Φ_*o*_ is the flux quantum and *μ*_*o*_ is the permeability constant. The *J*_*d*_ value that is found is roughly 0.3 GA/cm^2^ by using the following values of the penetration depth, *λ* = 105 nm and the coherence length, ξ = 2.7 nm^20^,^21^. Our estimation indicates that there is a considerable potential to further enhance flux pinning in (Ba,K)Fe_2_As_2_.

In this paper, we investigate the flux pinning of optimally doped (Ba,K)Fe_2_As_2_ under hydrostatic pressure. We demonstrate that hydrostatic pressure causes little change in *T*_*c*_, but leads to significant enhancement in flux pinning or *J*_*c*_ by a factor of 5 in both low and high fields in optimally doped Ba_0.6_K_0.4_Fe_2_As_2_ crystals. At 4.1 K, high pressure can significantly enhance *J*_*c*_ from 5 × 10^5 ^A/cm^2^ to nearly 10^6 ^A/cm^2^ at 2 T and from 2 × 10^5 ^A/cm^2^ to nearly 5.5 × 10^5 ^A/cm^2^ at 12 T. Our systematic analysis shows that the both *N*_*p*_ and *F*_*p*_ are increased by the pressure and contribute to strong pinning.

Figure 1 shows the temperature dependence of the magnetic moments for zero-field-cooled (ZFC) and field-cooled (FC) measurements at different pressures. *T*_*c*_ remains almost unchanged at different pressures. *T*_*c*_ ≈ 37.95 K was found at P = 0 GPa and P = 1 GPa. Similar results were also reported for Ba_0.6_K_0.4_Fe_2_As_2_ thin film^22^. Furthermore, a temperature independent magnetic moment at low temperatures was observed, along-with a small transition width, indicating the high quality of the crystals.

The field dependence of *J*_c_ at different temperatures (4.1, 16, and 24 K) and pressures (0 and 1.2 GPa), obtained from the magnetic hysteresis (*M*-*H)* curves by using Bean’s model, are shown in Fig. 2. Nearly five-fold *J*_c_ enhancement can be seen at 16 K and 24 K in both low and high fields at P = 1.2 GPa. It is noteworthy that *J*_*c*_ is enhanced for the Ba_0.6_K_0.4_Fe_2_As_2_ crystal at 1.2 GPa in both low and high fields. This has not been found with the other approaches for pinning enhancement reported so far. At 16 K and self-field, the *J*_*c*_ is 2 × 10^5 ^A/cm^2^ and it increases up to 6 × 10^5 ^A/cm^2^ under pressure of 1.2 Gpa, with as high a value as 3 × 10^5 ^A/cm^2^ retained at 12 T. At 24 K, *J*_*c*_ at zero field is 9 × 10^4 ^A/cm^2^ which increases to 2.5 × 10^5 ^A/cm^2^ at P = 1.2 Gpa, with the same value retained at 12 T. At 4.1 K, the *J*_*c*_ is nearly 1 × 10^6 ^A/cm^2^ at 2 T and 5 × 10^5 ^A/cm^2^ at 12 T under P = 1.2 GPa.

The pinning force (*F*_*p*_ = *J*_*c*_ × *B*) as a function of field at 8 K, 12 K, 24 K, and 28 K is shown in Fig. 3 23. At high fields and pressures, the *F*_*p*_ is found to be nearly 5 times higher at 8, 12, 24, and 28 K as compared to the corresponding value at P = 0 GPa, which agrees nicely with the *J*_*c*_ enhancement results. Figure 4 shows a comparison of *F*_*p*_ obtained in our Ba_0.6_K_0.4_Fe_2_As_2_ under pressure with those of several other low and high temperature superconducting materials^24^,^25^,^26^,^27^. The (Ba,K)Fe_2_As_2_ shows better in-field performance under pressure. Pressure can significantly improve *F*_*p*_ values to greater than 60 GN/m^3^ at *H* > 10 T, which are even superior to those of Nb_3_Sn and NbTi.

With respect to the *N*_*p*_, pressure can also increase the number of point pinning centres (point defects), which can suppress thermally activated flux creep, leading to *J*_*c*_ enhancement^12^. *N*_*p*_ is calculated by using the following equation^28^:


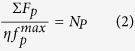


where ∑*F*_*p*_ is the accumulated pinning force density, 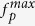 is the maximum elementary pinning force (*f*_*p*_), which is the interaction between a flux line and a single defect, and *η* is an efficiency factor. *η* = 1 corresponds to a plastic lattice, and the *η* value is otherwise 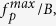 where *B* is the bulk modulus of the sample. We assume to a second order of approximation that the interaction between a flux line and a single defect is nearly the same under pressure. Therefore, we can use 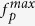 ≈ 3 × 10^−13 ^N for a similar superconductor (i.e., Ba122:Co) to estimate *N*_*p*_ 29. At 4.1 K, *N*_*p*_ ≈ 7.3 × 10^24 ^m^−3^ at P = 0 Gpa, which increases to *N*_*p*_ ≈ 1.2 × 10^25 ^m^−3^ for *P* = 1.2 GPa, while at 24 K, *N*_*p*_ ≈ 6.6 × 10^23 ^m^−3^ at P = 0 Gpa, which increases to *N*_*p*_ ≈ 3.8 × 10^24^/m^3^ for *P* = 1.2 GPa.

In order to examine if the pinning force enhancement is the major factor responsible for the observed *J*_*c*_ enhancement in our crystal under pressure, we have calculated the differences in the ratios of 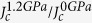 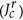 and 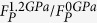 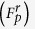 and plot the results in Fig. 5 as a function of field. Analysis of the 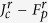 data, acquired at different temperatures, leads to values of nearly zero. This result indicates that *J*_*c*_ enhancement is only related to pinning force enhancement.

To examine whether the observed *J*_*c*_ enhancement is likely to be affected by volume change of the samples under high pressure, we have performed the following analysis. According to the Wentzel-Kramers-Brillouin (WKB) approximation, high pressure can affect the grain boundaries by reducing the tunnelling barrier width (*W)* and the tunnelling barrier height (*L)* for polycrystalline bulks, in accordance with the following simple mathematical expression^30^,^31^,^32^:


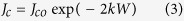


here *k* = (2 *mL*)^1/2^/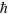 corresponds to the decay constant, where 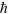 is the reduced Planck constant, and *J*_*c0*_ is the critical current density at 0 K and 0 T. The relative pressure dependence of *J*_*c*_ can be determined from Eq. (3) as^33^:


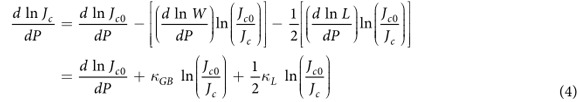


The reduction in the width and height of the grain boundaries can be written as 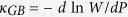 and 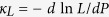, respectively.

We can use this model for the (Ba,K)Fe_2_As_2_ single crystals, by assuming to a first approximation that *κ*_GB_ and *κ*_L_ can be nearly equated to the average linear compressibility values *κ*_*a*_ = −*d*ln*a*/*dP* (*κ*_*a*_ ≈ 0.00318 GPa^−1^) and *κ*_*c*_ = −*d*ln*c*/*dP* (*κ*_*c*_ ≈ 0.00622 GPa^−1^), respectively, in the FeAs plane, where *a* and *c* are the in-plane and out-of-plane lattice parameters^34^. Consequently, Eq. (4) can be modified as


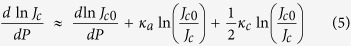


By using *J*_*c*_ ≈ 10^5 ^A/cm^2^ at 24 K and *J*_*c0*_ ≈ 10^6 ^A/cm^2^, 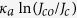 ≈ 0.0073 GPa^−1^ and (1/2 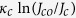) ≈ 0.0071 GPa^−1^, which contribute collectively not more than 2% of the experimentally obtained value, i.e., *d*ln*J*_c_/*dP* = 0.92 GPa^−1^ from the inset of Fig. 5. This illustrates that the source of the flux pinning under pressure is not the volume change.

The *J*_*c*_ value vs. reduced temperature (i.e. 1-*T*/*T*_*c*_) at 0 and 10 T under different pressures is shown in Fig. 6. The data points in different fields and pressures follow a power law description [i.e. *J*_*c*_ ∝ (1 − *T*/*T*_*c*_)^*β*^], where *β* is a critical exponent^35^,^36^,^37^. At specific fields, Ginzburg-Landau theory predicts distinct vortex pinning mechanisms, with different values of exponent *β*. For example *β* = 1 corresponds to non-interacting vortices and *β* ≥ 1.5 corresponds to the core pinning mechanism. Our value of *β* ~ 1.74 and 1.85 for zero field, and *β* ~ 1.20 and 1.43 at 10 T, at 0 and 1.2 GPa, respectively, reveal a robust dependence of *J*_*c*_ on pressure. The low *β* values at high pressure show the weak field dependences of *J*_*c*_ in contrast to its values at low pressure. Different values of exponent *β* have also been observed in MgB_2_ and yttrium barium copper oxide (YBCO)^38^,^39^.

The pinning mechanisms in Ba_0.6_K_0.4_Fe_2_As_2_ have been examined in the frame of collective pinning theory. Generally, core pinning comprises 1) *δl* pinning, which comes from spatial variation in the charge carrier mean free path, *l*, and 2) *δT*_*c*_ pinning due to randomly distributed spatial variation in *T*_c_.

Referring to the Griessen *et al.* approach:


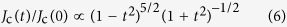


corresponds to 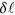 pinning, while


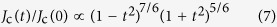


applies in the case of *δT*_*c*_ pinning, where *t* = *T/T*_*c*_ 40. Figure 7 shows almost perfect overlapping of the experimentally obtained *J*_c_ values and the theoretically expected variation in the *δl* pinning mechanism at 0.05 T. This is in agreement with the observation of little change in *T*_*c*_ under high pressure. We also observed similar results in BaFe_1.9_Ni_0.1_As_2_ and SiCl_4_ doped MgB_2_ 41,42. Furthermore, *δl* pinning has also been reported in FeTe_0.7_Se_0.3_ crystals^43^.

In conclusion, we have systematically examined the flux pinning in optimally doped Ba_0.6_K_0.4_Fe_2_As_2_ crystal under hydrostatic pressure, analyzing the critical current density that was determined experimentally. We have demonstrated that strong flux pinning in both low and high fields can be achieved by improving the pinning force under pressure. The pressure of 1.2 GPa improved the *F*_*p*_ by nearly 5 times at 8, 12, 24, and 28 K, which can increase *J*_*c*_ by nearly two-fold at 4.1 K and five-fold at 16 K and 24 K over a wide range of fields. This study also demonstrates that the performance of an optimally doped superconductor in both low and high fields can also be further enhanced by pressure.

## Experimental

High quality 122 crystals were grown by the flux method. The pure elements Ba, K, Fe, As, and Sn were mixed in a mol ratio of Ba_1−*x*_K_*x*_Fe_2_As_2_:Sn = 1:45–50. A crucible with a lid was used to control the evaporation loss of potassium along with that of arsenic during growth. The crucible was sealed in a quartz ampoule filled with Ar and loaded into a box furnace^15^. The *M*-*H* loops at different temperatures and pressures and the temperature dependence of the magnetic moments were measured on a Quantum Design Physical Properties Measurement System (QD PPMS 14 T) by using the Vibrating Sample Magnetometer (VSM) option. We used an HMD high pressure cell and Daphne 7373 oil as the medium for applying hydrostatic pressure on our samples. Further details can be found in pressure cell manual i.e. Quantum Design (QD) High Pressure Cell User Manual for use with the QD VSM, No. CC-Spr-Φ8.5D-MC4. The magnetic fields were applied parallel (H//ab) to the ab-plane of the samples.

## Additional Information

**How to cite this article**: Shabbir, B. *et al.* Study of flux pinning mechanism under hydrostatic pressure in optimally doped (Ba,K)Fe_2_As_2_ single crystals. *Sci. Rep.* **6**, 23044; doi: 10.1038/srep23044 (2016).

## Figures and Tables

**Figure 1:**
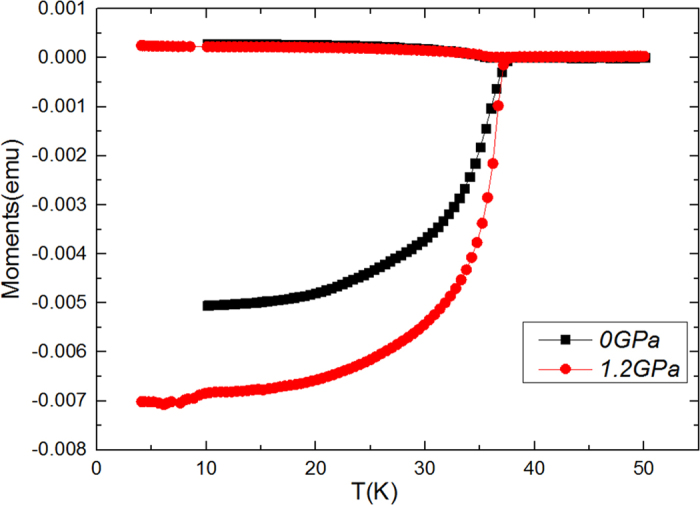
Magnetic moments vs. temperature at P = 0 GPa and P = 1.2 GPa. *T*_*c*_ remains almost unchanged at different pressures. *T*_*c*_ ≈ 37.95 K was found at P = 0 GPa and P = 1 GPa.

**Figure 2:**
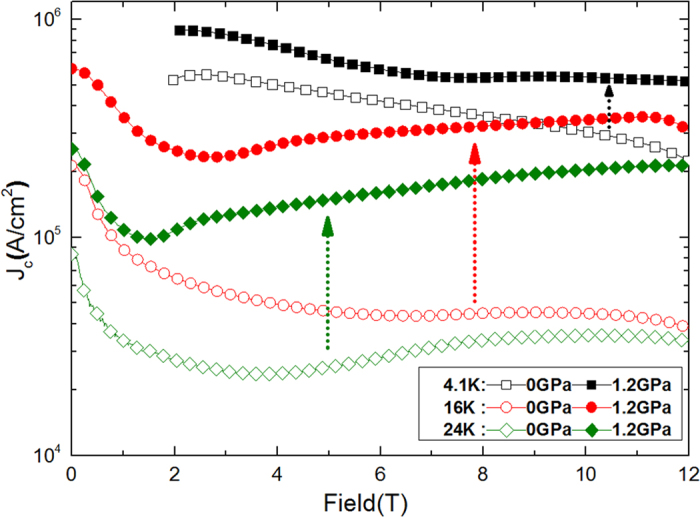
*J*_*c*_ as a function of field at *P* = 0 and 1.2 GPa at 4.1, 16, and 24 K. *J*_*c*_ is improved in both low and high fields and nearly five-fold *J*_c_ enhancement can be seen at 16 K and 24 K in both low and high fields at P = 1.2GPa.

**Figure 3:**
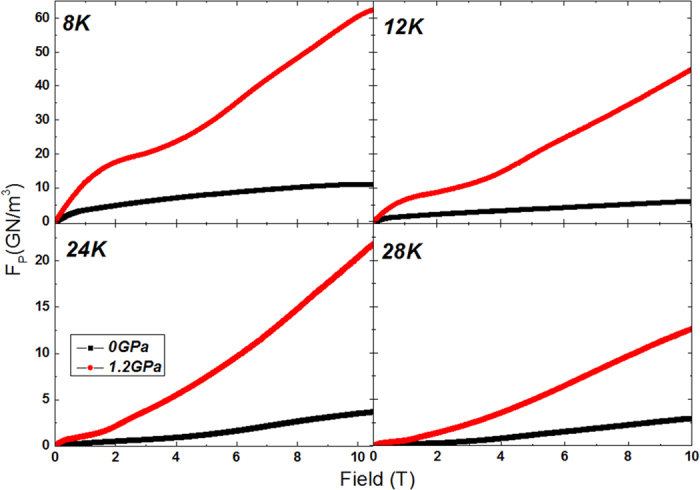
*F*_*p*_ versus field at 8, 12, 24, and 28 K at different pressures. At high fields and pressures, the *F*_*p*_ is found to be nearly 5 times higher at 8, 12, 24, and 28 K as compared to the corresponding value at P = 0 GPa, which agrees nicely with the *J*_*c*_ enhancement results.

**Figure 4:**
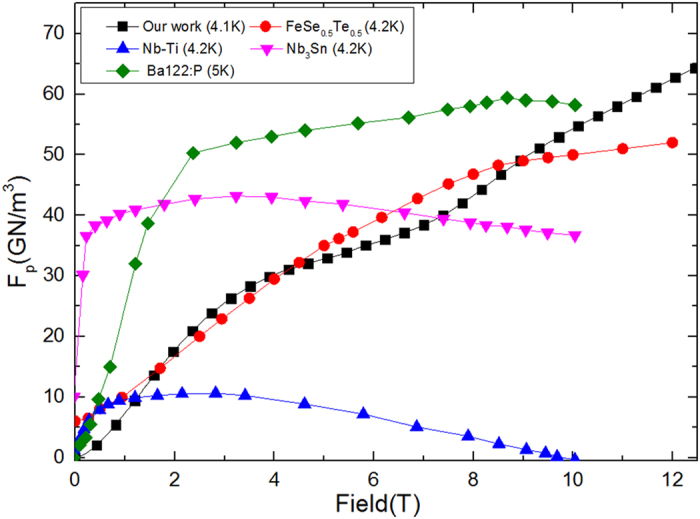
Comparison of *F*_*p*_ for different superconductors. Pressure can significantly improve *F*_*p*_ values to greater than 60 GN/m^3^ at *H* > 10 T, which are even superior to those of Nb_3_Sn and NbTi.

**Figure 5:**
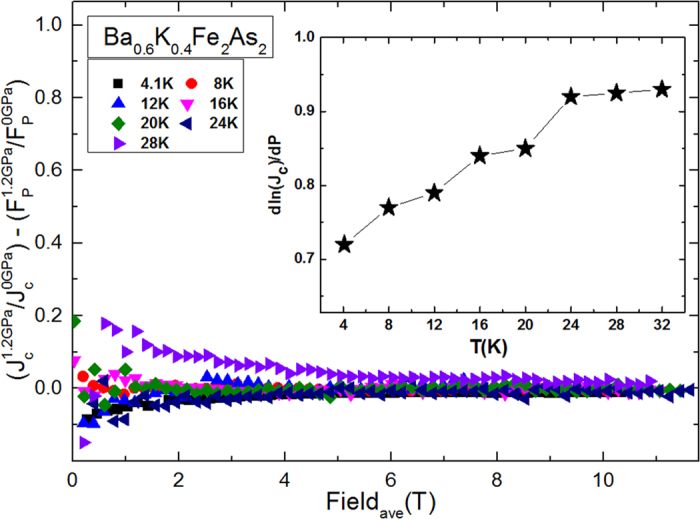
*J*_*c*_−*F*_*p*_ ratios at *P* = 1.2 GPa and *P* = 0 GPa. The relative change of ln*J*_*c*_ with pressure as a function of *T* is given in the inset. Analysis of the *J*_*c*_-*F*_*p*_ ratios, acquired at different temperatures, leads to values of nearly zero. This result indicates that *J*_*c*_ enhancement is only related to pinning force enhancement.

**Figure 6:**
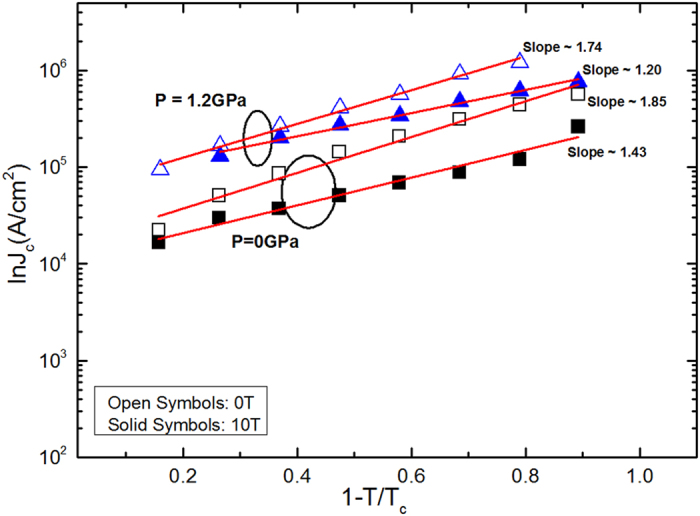
ln*J*_*c*_ versus reduced temperature at different fields and pressures. The low *β* values at high pressure show the weak field dependences of *J*_*c*_ in contrast to its values at low pressure.

**Figure 7:**
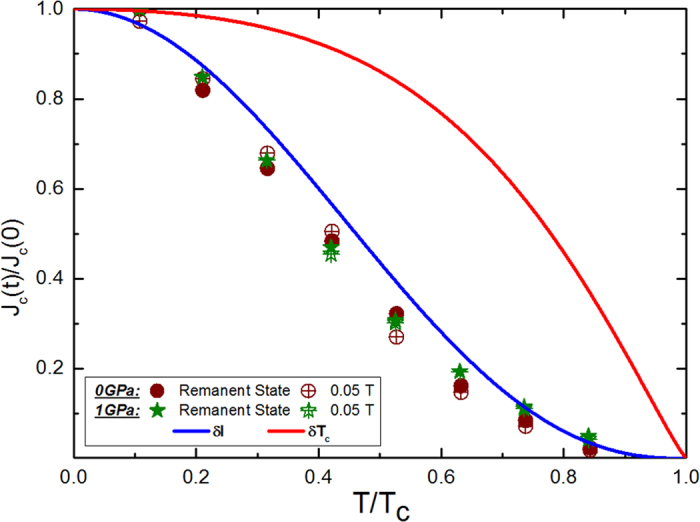
*J*_*c*_ as a function of *T/T*_*c*_. Experimental data points are in good agreement with *δl* pinning.
